# Draft Genome Sequence of a Tropical Freshwater Cyanobacterium, *Limnothrix* sp. Strain P13C2

**DOI:** 10.1128/genomeA.01117-16

**Published:** 2016-10-20

**Authors:** Boon Fei Tan, Shu Harn Te, Karina Yew-Hoong Gin, Janelle R. Thompson

**Affiliations:** a Centre for Environmental Sensing and Modelling, Singapore-MIT Alliance for Research and Technology, Singapore; b NUS Environmental Research Institute, National University of Singapore, Singapore; c Department of Civil and Environmental Engineering, National University of Singapore, Singapore

## Abstract

A nonaxenic unialgal culture of *Limnothrix* sp. strain P13C2 was obtained through multiple subculturing of an inoculum obtained from a tropical freshwater lake. Here, we report the genome of P13C2 of 4.6 Mbp, extracted from the metagenome of this coculture.

## GENOME ANNOUNCEMENT

*Limnothrix* is a ubiquitous cyanobacterium commonly found in many freshwater and marine environments (1, 2). Research conducted on this genus is limited because the organism is rarely the most abundant species during bloom events—they have been found only occasionally to codominate an algal bloom with other cyanobacterial species (3). *Limnothrix* spp. were not known to be toxin producers in the past until recent studies demonstrated that they cause toxicological effects in aquatic organisms, with symptoms similar to neurotoxin exposure (4, 5). Phylogenetic analysis based on 16S rRNA gene sequences showed that members that have been previously classified as *Limnothrix* are polyphyletic, forming two clades with similarity lower than 90% (6); although the difference at the genomic scale for these strains is unknown.

A filamentous cyanobacterium identified as *Limnothrix* sp. strain P13C2 based on 16S rRNA gene and morphology was isolated and cultivated using methods described previously (7). The nonaxenic unialgal culture of strain P13C2 used in this study was cultivated at room temperature for 2 weeks, after which total DNA was isolated and sequenced using the Illumina HiSeq 2000 platform as previously described (7). Adaptor and bar code sequences were removed from all reads using BBDuk of the BBTool packages (https://sourceforge.net/projects/bbmap/), and the reads were *de novo* assembled into scaffolds using CLC Genomics Workbench V8 with default settings. All quality-controlled unassembled reads were subjected to BLASTX searches against the NCBI NR database using DIAMOND (8) and taxonomic assignment using MEGAN 6 (9), revealing that the metagenome was represented with >50% of reads being assigned to *Cyanobacteria*. The genome of P13C2 was extracted from the minimetagenome using MetaBAT (10), and genome completeness and contamination were determined using checkM (11). The genome was subsequently annotated using the RAST platform (12).

The genome of P13C2 is >99% complete and contained zero sequence contaminant, determined using checkM (11). The genome of 4.6 Mbp is contained in 32 scaffolds (*N*_50_ 192 kbp) with 55% GC content. A single copy the 16S rRNA gene is 99% to 100% identical to those in several *Limnothrix* spp. (e.g., *Limnothrix planktonica* CHAB763; JQ004026.2). Comparison to genomes of other *Limnothrix* spp. was not conducted as these genomes were unavailable in the JGI IMG (13) and NCBI genome databases at the time of this analysis. Certain *Limnothrix* spp. strains have been previously found to produce toxin (4, 5), although the gene encoding hepatoxin (e.g., microcystin, saixtoxin), as well as off-flavor compounds (e.g., geosmin and 2-MIB), were not detected in the P13C2 genome. The genome encodes various pathways for central carbohydrate metabolism, the citric acid cycle, pentose-phosphate pathway, Entner-Doudoroff pathway, and glycolysis, thus suggesting that P13C2 may be physiologically versatile. The genome lacks the gene encoding nitrogenase, suggesting that P13C2 is incapable of nitrogen fixation. The organism likely obtains its nitrogen source through import of nitrogenous compounds using cyanate ABC transporter, nitrate ABC transporter, and ammonium transporter, whereby genes encoding these permeases were detected in the draft genome. In light of current knowledge gaps, additional genomic information on *Limnothrix* sp. is essential for taxonomic revision and evaluation of their potential public health risk.

### Accession number(s).

This whole-genome shotgun project has been deposited at DDBJ/ENA/GenBank under the accession number MBRF00000000. The version described in this paper is version MBRF01000000.
